# Swallowing and swallowing‐breathing interaction as predictors of intubation in Guillain‐Barré syndrome

**DOI:** 10.1002/brb3.611

**Published:** 2016-12-20

**Authors:** Adam Ogna, Helene Prigent, Michele Lejaille, Patricia Samb, Tarek Sharshar, Djillali Annane, Frederic Lofaso, David Orlikowski

**Affiliations:** ^1^ Service de Réanimation médicale et unité de ventilation à domicileHôpital Raymond PoincaréGarchesFrance; ^2^ Service de Physiologie‐Explorations FonctionnellesHôpital Raymond PoincaréGarchesFrance; ^3^ INSERM CIC 14.29Hôpital Raymond PoincaréGarchesFrance; ^4^ Unité de Recherche Clinique Paris OuestDépartement d'Information Hospitalière et de Santé PubliqueHôpital Ambroise‐ParéBoulogneFrance

**Keywords:** dysphagia, Guillain‐Barré syndrome, ICU, intubation, tongue strength, vital capacity

## Abstract

**Background:**

Bulbar weakness and respiratory impairment have been associated with increased morbidity in retrospective studies of Guillain‐Barré syndrome (GBS) patients. The aim of this study was to prospectively explore the relationship between subclinical swallowing impairment, respiratory function parameters, the necessity to intubate patients and the development of early postintubation pneumonia in patients with GBS in the intensive care unit (ICU)**.**

**Methods:**

Respiratory, swallowing, and tongue strength parameters were measured in 30 consecutive adults (51.7 ± 18.1 years old), hospitalized for GBS in the ICU of a teaching hospital. Twenty healthy volunteers were recruited as a control group. The primary outcomes were intubation and pneumonia during the ICU stay.

**Results:**

Nineteen patients (65.5%) had piecemeal swallowing, and 19 (65.5%) had impaired breathing‐swallowing interaction, of which, respectively, 47.4% and 52.6% had a clinically apparent swallowing impairment. Swallowing impairment was associated with lower values of respiratory function, but not with peripheral motor weakness. Tongue protrusion strength was correlated with respiratory parameters and swallowing impairment. Ten patients were intubated and six developed pneumonia. Age, BMI, severe axial involvement, respiratory parameters (vital capacity and respiratory muscle strength), tongue protrusion strength, and clinical swallowing impairment were predictors of intubation.

**Conclusions:**

Swallowing impairment was present early after ICU admission in over 80% of patients and was an important predictor of intubation. A systematic clinical evaluation of swallowing should be carried out, eventually combined with an evaluation of tongue protrusion strength, along with the usual assessment of neurological and respiratory function, to determine the severity of the GBS.

## 1 Introduction

Guillain Barré Syndrome (GBS) is an immune‐mediated, acute, rapidly progressive neurologic disease which affects the peripheral nervous system. It is the first disease‐related cause of extensive paralysis in industrialized countries, with an incidence of 1–2 cases per 100,000 inhabitants (McGrogan, Madle, Seaman, & de Vries, 2009; Sejvar, Baughman, Wise, & Morgan, 2011). GBS is characterized by stereotypical symmetrical, centripetal progressive muscle weakness of variable severity and progression. One third of patients require mechanical ventilation at some point during the disease. Impairment of respiratory muscles and swallowing are the main factors that influence morbidity and mortality and therefore require close monitoring in the intensive care unit (ICU) (Rajabally & Uncini, 2012; Ropper, 1986; Ropper & Kehne, 1985).

Several predictors of need for mechanical ventilation have been identified in previous studies, such as rapidly progressing muscle weakness, inability to cough, bulbar weakness, and a rapid decrease in respiratory function (Chevrolet & Deleamont, 1991; Lawn, Fletcher, Henderson, Wolter, & Wijdicks, 2001; Sharshar, Chevret, Bourdain, & Raphael, 2003; Walgaard et al., 2010). The optimal time for intubation is difficult to determine and is based on a combination of clinical criteria, blood gas abnormalities, and respiratory assessment during repeated measurements (Prigent, Orlikowski et al., 2012; Ropper, 1994; Wijdicks & Borel, 1998). Bulbar‐related impairments can lead to aspiration pneumonia, particularly if the patient's capacity to cough is reduced. We recently showed that 75% of ventilated patients with GBS developed pneumonia, mostly during the first 5 days of intubation, with a bacteriological profile consistent with inhalation (Orlikowski et al., 2006). These findings suggest that bulbar involvement is often detected too late to prevent swallowing‐related complications such as bronchial congestion or respiratory distress caused by aspiration.

Subclinical swallowing disorders may be present in patients with neuromuscular disorders, particularly in patients with severe respiratory impairment (Terzi et al., 2007). In a previous study of patients with Guillain‐Barré syndrome, tongue weakness was present early in the course of the disease, especially in patients who subsequently required mechanical ventilation, suggesting that a reduction in tongue protrusion strength could be an indicator of subclinical swallowing impairment (Orlikowski et al., 2009).

The aim of this study was to explore the impact of bulbar dysfunction and respiratory function parameters on respiratory failure necessitating intubation and on the development of early postintubation pneumonia in patients with Guillain‐Barré syndrome admitted to ICU, and to evaluate whether tongue strength could be an indicator of subclinical bulbar dysfunction.

## 2 Material and methods

### 2.1 Study population

Consecutive adults admitted to the Intensive Care Unit following onset of Guillan‐Barré syndrome were screened for inclusion. Patients requiring intubation on admission were excluded. Respiratory function, swallowing capacity, and tongue strength were evaluated at inclusion. Twenty healthy volunteers were recruited as a control group for swallowing and tongue strength parameters.

The study was approved by the local ethics committee (Comité de Protection des Personnes Ile de France XI). All participants gave written informed consent. ClinicalTrials.gov Identifier: NCT01024088.

### 2.2 Swallowing and tongue strength assessment

Swallowing was assessed clinically by a bedside drinking test (100 ml of water). The swallowing maneuver was also evaluated instrumentally: activity of the swallowing muscles was recorded using surface electromyography, laryngeal movements were recorded with an accelerometer and respiration was recorded simultaneously by inductance plethysmography, as previously described (Terzi et al., 2007). Data were recorded on an analog‐digital acquisition system (MP100; Biopac System, Santa Barbara, CA, USA) and analyzed by measuring the duration of the oro‐pharyngeal phase and the number of swallowing movements with increasing fluid quantities (5, 10, and 20 ml). For each fluid quantity, the mean value of 4 trials was calculated. The test was interrupted if signs of swallowing impairment were present. The results were interpreted according to two components of swallowing: “piecemeal deglutition”, considered as normal if the subject could swallow 20 ml in <2 swallows within 8 s; and “breathing‐swallowing interaction”, which was considered normal if the patient resumed breathing with expiration in ≥3 of the 4 trials (Aydogdu et al., 2015; Ertekin, Aydogdu, & Yuceyar, 1996). The global instrumental swallowing evaluation was considered as normal if both components were normal.

Tongue protrusion strength was measured using a previously described method (Orlikowski et al., 2009). Briefly, an impression of the mandibular teeth and floor of the mouth (Optosil P; Optosil Xantropen, Dormagen, Germany) was secured to a lingual force transducer (Neuro Logic Inc., Lawrence, KS, USA), which measured the force applied by the tongue, with a linear response signal from 50 to 1500 g. Data were amplified and recorded using an analog‐digital system (MP100; Biopac System).

### 2.3 Clinical and respiratory measurements

Motor impairment was assessed using the Hughes functional grading scale and the Medical Research Council (MRC) sum score. The Hughes scale ranges from 0 (normal) to 6 (death), with 4 signifying an inability to walk >5 m and 5 the need for mechanical respiratory support (Hughes, Newsom‐Davis, Perkin, & Pierce, 1978). The MRC sum score was computed as the sum of the strength scores (rated from 0 to 5) of six different muscle groups measured bilaterally, resulting in a score ranging from 0 (tetraplegic) to 60 (normal) (Kleyweg, van der Meche, & Schmitz, 1991).

Slow inspiratory VC was measured in triplicate with a spirometer (Morgan, UK), following standard guidelines. To determine maximal inspiratory pressure (MIP) at the residual volume and maximal expiratory pressure (MEP) at total lung capacity, patients breathed into a mouthpiece connected to a manometer. The maneuvers were repeated at least three times or until two identical readings were obtained. For each maneuver, the best result was analyzed.

### 2.4 Statistical analysis

Statistical analysis was conducted using SAS 9.3. statistical software (SAS Institute Inc, Cary, NC, USA). Chi square and Wilcoxon tests were used to compare subgroups. Univariate logistic regression analysis was performed to identify predictors of intubation and to estimate the strength of association based on the odds ratio (OR). It was not possible to fit a multivariate model, because of the large number of candidate variables compared to few outcomes and the presence of multiple correlations between the predictors. Statistical significance was established at *p *< .05.

## 3 Results

### 3.1 Study population

Thirty patients were included; they all met the Asbury criteria for defining Guillain‐Barré syndrome. Mean age was 51.7 ± 18.1 years, 28 patients (93%) had a Hughes score of 4. The time between onset of the GBS symptoms and ICU admission was 4.6 ± 4.6 days. All patients received an immune‐modulating treatment, 23 (77%) received intravenous immunoglobulins and 9 (30%) underwent plasma‐exchange. The mean time between onset of the GBS symptoms and first treatment was 5.6 ± 4.6 days. The characteristics of the study population are further detailed in Table 1.

**Table 1:** Characteristics of the study population

Parameter	Patients with GBS	Healthy controls	*p*
N	30	20	
Men, %	53.3	50	NS
Age (year)	57 (32–65)	39 (30–49.5)	.023
BMI (kg/m^2^)	26.3 (22.5–30.4)	22.5 (21.8–24.4)	.032
Muscle strength			
Time since impairment onset (d)	3 (3–5)	–	
MRC sum score	35 (30–42)	–	
Facial paralysis, %	36.7	–	
Inability to lift the head, %	16.7	–	
Tongue strength (g)	598 (400.5–1006)	885 (784.5–1011)	.024
Respiratory parameters			
Sitting VC (% pred)	67 (48–79)	95.5 (92–104)	<.0001
MIP (cmH_2_O)	28 (21–49)	93.5 (73–110)	<.0001
MEP (cmH_2_O)	28 (20–51)	77 (49.5–102)	.0003
Peak cough flow (l/min)	248 (163–324)	443 (370–549)	<.0001
Swallowing			
Clinical swallowing impairment, %	36.7	–	
Instrumental swallowing assessment:			
5* *ml	*N =* 29	*N =* 20	
Duration of swallow (s)	2.5 (1.3–3.6)	1.3 (1.2–1.5)	.004
Number of swallows	1.25 (1–1.75)	1 (1–1)	.0004
% expiratory breath resumption	75 (40–100)	100 (79–100)	.002
10 ml	*N =* 22	*N *= 20	
Duration of swallow (s)	3 (1.5–5)	1.3 (1.2–1.5)	.0005
Number of swallows	1.5 (1–2.25)	1 (1–1)	.002
% expiratory breath resumption	83 (50–100)	100 (100–100)	.001
20 ml	*N =* 18	*N =* 20	
Duration of swallow (s)	3.6 (2.4–5.3)	1.7 (1.4–3)	.014
Number of swallows	1.75 (1.25–2.5)	1 (1–1.5)	.005
% expiratory breath resumption	75 (50–83)	100 (75–100)	.006

BMI, body mass index; MRC, Medical Research Council; VC, vital capacity; % pred, percentage of the predicted value; MIP, maximal inspiratory pressure; MEP, maximal expiratory pressure.

Values are expressed as medians (IQR) unless otherwise specified.

### 3.2 Swallowing assessment

At ICU admission, 11 patients (37%) had a clinically evident swallowing impairment. During the ICU stay, 15 (50%) patients developed clinical swallowing disorders which required the insertion of a gastric tube.

Twenty‐nine of the 30 patients with GBS underwent the instrumental swallowing evaluation. All swallowing parameters were impaired: swallowing times were longer, even for small volumes; there was a greater number of sips for each swallowing volume, and a smaller percentage of patients resumed respiration with expiration compared to the healthy controls (Table 1). In 11 patients (37%), the instrumental swallowing test was interrupted before reaching 20 ml because of signs of inhalation. Nineteen patients (65.5%) showed pathological piecemeal swallowing, and 19 patients (65.5%) had impaired breathing‐swallowing interaction. Only five of the patients (17.2%) were considered to have normal swallowing at ICU admission based on these criteria. 47.4% of the patients with piecemeal deglutition and 52.6% of those with an impaired breathing‐swallowing interaction had a clinically apparent swallowing impairment.

### 3.3 Swallowing, respiratory, and neurological impairment

Patients with an abnormal instrumental swallowing test had a significantly lower VC than participants with a normal assessment (61.0 ± 17.3% vs. 86.6 ± 19.7%, *p *= .01). This difference was found for both the patients with piecemeal deglutition (VC 58.8 ± 18.4% vs. 77.9 ± 17.1%, *p *= .02) and for those with impaired breathing‐swallowing interaction (VC 59.5 ± 17.9% vs. 76.7 ± 19.5%, *p *= .03).

There was no significant difference between the degree of motor impairment of patients with a normal or abnormal instrumental swallowing test (MRC score 37.2 ± 12.4 vs. 32.6 ± 10.4 and prevalence of axial muscle weakness 80% vs. 70.8%).

### 3.4 Tongue protrusion strength

Tongue protrusion strength was correlated with respiratory parameters (*r *= .383, *p *= .04 for VC and *r *= .428, *p *= .03 for MIP) and with the presence of a swallowing impairment, assessed both clinically and instrumentally: patients with a clinically evident swallowing impairment had significantly lower tongue protrusion strength than those without swallowing impairment (493.2 ± 321.8 vs. 802.0 ± 317.5 g, *p *= .03). A similar result was observed for both piecemeal deglutition (tongue protrusion strength 548.6 ± 337.1 vs. 856.8 ± 293.6 g, *p *= .04) and impaired breathing‐swallowing interaction (562.9 ± 345.0 vs. 830.9 ± 304.1 g, *p *= .05).

### 3.5 Intubation and postintubation pneumonia

Ten patients (33%) were intubated; mean time between ICU admission and intubation was 4.4 ± 4.4 days. Mean duration of ventilation was 48.7 ± 83.9 days. Six (60%) out of the 10 mechanically ventilated patients developed pneumonia a mean of 9.7 ± 5.9 days following ICU admission. None of the nonintubated patients developed pneumonia.

Age, BMI, inability to lift the head as a marker of severe proximal and axial involvement, respiratory parameters (reduction in vital capacity and respiratory muscle strength), tongue protrusion strength and clinical swallowing impairment were predictors of intubation (Tables 2 and 3).

**Table 2:** Comparison of intubated and nonintubated patients

Parameter	Not intubated	Intubated	*p*
N	20	10	
Men, %	60	40	NS
Age (year)	62 (45.5–72)	38.5 (27–58)	.029
BMI (kg/m^2^)	27.4 (24.8–31)	22.5 (21.3–27.5)	.040
Muscle strength			
Time since impairment onset (d)	3.5 (3–5.5)	3 (3–5)	NS
MRC sum score	36 (30–44)	31.5 (27–37)	.168
Facial paralysis, %	30	50	NS
Inability to lift the head, %	0	50	.002
Tongue strength (g)	803 (425–1040)	355 (125–704)	.027
Respiratory parameters			
Sitting VC (% pred)	75 (55–85)	53 (37–70)	.031
Supine VC (% pred)	74.5 (55.5–91)	50.5 (35–72)	.037
MIP (cmH_2_O)	34 (25.5–52.5)	21 (21–28)	.028
MEP (cmH_2_O)	37.5 (21–67)	20 (15–28)	.030
Peak cough flow (l/min)	248 (164–324)	219.5 (108–322)	NS
Swallowing			
Clinical swallowing impairment, %	20	70	.015
Instrumental swallowing impairment, %	78.9	90	NS
Piecemeal deglutition, %	57.9	80	NS
Breathing‐swallowing interaction, %	57.9	80	NS

BMI, body mass index; MRC, Medical Research Council; VC, vital capacity; % pred, percentage of the predicted value; MIP, maximal inspiratory pressure; MEP, maximal expiratory pressure.

Values are expressed as medians (IQR) unless otherwise specified.

**Table 3:** Predictors of intubation (univariate analysis)

Parameter	OR (95% CI)	*p*
Age (year)	0.95 (0.91; 0.99)	.03
BMI (kg/m^2^)	0.82 (0.67; 1.00)	.04
Muscle strength		
Time since impairment onset (d)	1.07 (0.90; 1.28)	NS
Time since impairment onset <7 days	0.26 (0.04; 1.90)	NS
MRC sum score	0.95 (0.88; 1.02)	.17
Inability to lift the head	44.3 (3.9; 500)	<.001
Tongue strength (g)	0.997 (0.99; 1.00)	.03
Respiratory parameters		
VC (% pred)	0.95 (0.91; 1.00)	.03
VC <60%	4.50 (0.89; 22.7)	.11
MIP (cmH_2_O)	0.92 (0.84; 1.00)	.03
MEP (cmH_2_O)	0.94 (0.88; 1.00)	.03
Peak cough flow (l/min)	0.99 (0.98; 1.00)	.16
Swallowing		
Clinical swallowing impairment	9.33 (1.64; 53.2)	.007
Instrumental swallowing impairment	0.42 (0.04;4.33)	NS

BMI, body mass index; MRC, Medical Research Council; VC, vital capacity; % pred, percentage of the predicted value; MIP, maximal inspiratory pressure; MEP, maximal expiratory pressure.

Values are expressed as medians (IQR) unless otherwise specified.

There were no differences between the clinical and functional parameters of patients who developed pneumonia and those who did not.

## 4 Discussion

In this study, we observed a swallowing impairment occurring early in the course of Guillain‐Barré syndrome, in parallel with the decrease in respiratory function, as previously described in other neuromuscular diseases (Terzi et al., 2007). More than 80% of patients had impaired swallowing on admission to ICU, and clinical swallowing impairment was one of the strongest predictors of intubation.

According to current understanding, two components of impaired swallowing can be differentiated: 1) fragmentation of the bolus into several small volumes, termed “piecemeal deglutition”, which is caused by muscle weakness and 2) impairment of breathing‐swallowing interaction with a decrease in the percentage of swallows followed by physiological expiration, which is caused by respiratory failure. Both these components were present in our GBS population early in the course of the disease, and both were associated with impaired respiratory function, in line with previous descriptions of chronic neuromuscular diseases (Terzi et al., 2007). The interactions between respiratory and swallowing impairments seem to be complex in GBS. The diaphragm and the genioglossus muscle are regulated by common mechanisms, suggesting that bulbar muscle function and respiratory regulation might be intimately correlated (Carlson, Carley, Onal, Lopata, & Basner, 1994; Onal, Lopata, & O'Connor, 1981). Bulbar involvement can thus lead to respiratory failure and should be identified early. Our results showed that, along with clinical and instrumental swallowing evaluations, tongue strength was a valid proxy for the evaluation of bulbar muscle function. This has previously been described in a smaller group of patients with severe GBS (Orlikowski et al., 2009).

Both clinical and physiological parameters were significantly associated with intubation. Clinical factors included severe axial muscle weakness and swallowing impairment on admission, and physiological parameters included a reduction in vital capacity, respiratory muscle strength, and tongue protrusion strength. Most of these factors have previously been identified in retrospective studies (Chevrolet & Deleamont, 1991; Lawn et al., 2001; Sharshar et al., 2003) and several have been shown to progress in parallel with the disease. In our population, swallowing impairment on admission and axial weakness emerged as the two strongest predictors of intubation.

In a pooled analysis of retrospective studies, Walgaard et al. (2010) found a similar predictive value of bulbar weakness, however, respiratory function was not considered. This is a major limitation since neurological features, such as impossibility to raise the head and bulbar involvement, have been shown to predict intubation if respiratory function is not considered, however, once VC is assessed, these features are no longer predictive of intubation (Sharshar et al., 2003).

It is thus essential to evaluate bulbar muscle impairment, however, it is not simple. Other than the basic clinical evaluation, there are no simple tools to evaluate the severity of bulbar disorders at the bedside and videofluroscopic swallowing evaluation is difficult to perform in ICU patients (Chen, Donofrio, Frederick, Ott, & Pikna, 1996). The bedside clinical swallowing evaluation has been criticized for its low accuracy and it has been reported that even the most experienced clinicians fail to identify about 40–50% of aspirating patients during a bedside examination (Ertekin et al., 1996). In line with these observations, 37% of the patients in this study were found to have a swallowing impairment following the clinical evaluation, whereas the instrumental evaluation showed that more than 80% of the patients actually had a swallowing impairment.

Although the instrumental swallowing evaluation was more sensitive for the identification of swallowing impairment, it did not detect patients at risk of subsequent intubation better than the standardized clinical assessment. This is in accordance with the hypothesis that impairment of breathing‐swallowing interaction and bolus fragmentation are the direct consequences of respiratory failure, which is probably the main factor which leads to intubation. This is supported by the improvement of swallowing parameters we previously obtained treating respiratory failure with invasive mechanical ventilation in patients with neuromuscular disorders (Prigent, Lejaille, et al., 2012; Terzi et al., 2010), and with noninvasive ventilation in patients with COPD (Terzi et al., 2014). This study extends the findings of previous reports of impaired breathing‐swallowing interaction in patients with COPD (Gross, Atwood, Ross, Olszewski, & Eichhorn, 2009; Terzi et al., 2014) and chronic neuromuscular disorders (Terzi et al., 2007), to patients with GBS, allowing us to infer that respiratory failure is the common cause of these swallowing disorders. The early detection of swallowing disorders may thus allow the swallowing pattern to be improved, potentially preventing possible complications such as aspiration. However, a systematic evaluation of swallowing and swallowing‐breathing interaction using instrumented methods does not seem to be justified in daily clinical practice. Our results showed that the assessment of tongue strength and a standardized clinical evaluation of swallowing provide a bedside appreciation of bulbar involvement, and should therefore be part of the routine assessment of patients with GBS. Combined with respiratory function, the assessment of tongue protrusion strength may be useful to identify patients at risk of respiratory failure and may help to better define the optimal time to intubate. It should, however, be stressed that no cut‐off value has yet been defined for the interpretation of tongue strength, therefore further studies are necessary before this can be applied in daily clinical practice.

In line with previous observations, one third of the patients required intubation and mechanical ventilation, 60% of whom then developed pneumonia (Orlikowski et al., 2006; Ropper & Kehne, 1985). However, since the subgroup of ventilated patients was small, we could not fully analyze differences in clinical or physiological parameters between patients who developed pneumonia and those who did not. Another important limitation of this study was that factors previously associated with the risk of mechanical ventilation, such as inability to lift the head, may have influenced the decision to intubate the patient, leading to an overestimation of their real predictive role.

In conclusion, the results of our study suggest that swallowing impairments occur early after ICU admission and are an important predictor of intubation in patients with Guillain‐Barré syndrome. It is thus crucial to carry out a clinical evaluation of swallowing in combination with the usual clinical and respiratory evaluations, to assess the severity of the condition. Since there are no simple tools for the bedside detection of swallowing impairment, a standardized clinical evaluation combined with the assessment of tongue protrusion strength can identify bulbar involvement, and may identify patients at risk of respiratory failure.

## Conflict of Interest

The authors declare that they have no conflict of interest.
